# Fluid‐Infiltrated Metalens‐Driven Reconfigurable Intelligent Surfaces for Optical Wireless Communications

**DOI:** 10.1002/advs.202406690

**Published:** 2024-09-28

**Authors:** Ramna Khalid, Jaekyung Kim, Nasir Mahmood, Humberto Cabrera, Muhammad Qasim Mehmood, Aaron Danner, Muhammad Zubair, Junsuk Rho

**Affiliations:** ^1^ MicroNano Lab Department of Electrical Engineering Information Technology University of the Punjab (ITU) Lahore 54000 Pakistan; ^2^ SZCU‐ITU Joint International MetaCenter for Advanced Photonics and Electronics Information Technology University of the Punjab (ITU) Lahore 54000 Pakistan; ^3^ Department of Mechanical Engineering Pohang University of Science and Technology (POSTECH) Pohang 37673 Republic of Korea; ^4^ SZCU‐ITU Joint International MetaCenter for Advanced Photonics and Electronics Suzhou City University Suzhou 215104 China; ^5^ MLab, STI Unit The Abdus Salam International Centre for Theoretical Physics Trieste 34151 Italy; ^6^ Department of Electrical and Computer Engineering National University of Singapore Singapore 117583 Singapore; ^7^ Department of Chemical Engineering Pohang University of Science and Technology (POSTECH) Pohang 37673 Republic of Korea; ^8^ Department of Electrical Engineering Pohang University of Science and Technology (POSTECH) Pohang 37673 Republic of Korea; ^9^ POSCO‐POSTECH‐RIST Convergence Research Center for Flat Optics and Metaphotonics Pohang 37673 Republic of Korea; ^10^ National Institute of Nanomaterials Technology (NINT) Pohang 37673 Republic of Korea

**Keywords:** fluid‐infiltration, intelligent metasurface, optical communication, reconfigurable metalens, varifocal metalens

## Abstract

A reconfigurable intelligent surface (RIS), a leading‐edge technology, represents a new paradigm for adaptive control of electromagnetic waves between a source and a user. While RIS technology has proven effective in manipulating radio frequency waves using passive elements such as diodes and MEMS, its application in the optical domain is challenging. The main difficulty lies in meeting key performance indicators, with the most critical being accurate and self‐adjusting positioning. This work presents an alternative RIS design methodology driven by an all‐silicon structure and fluid infiltration, to achieve real‐time control of focal length toward a designated user, thereby enabling secure data transmission. To validate the concept, both numerical simulations and experimental investigations of the RIS design methodology are conducted to demonstrate the performance of fluid‐infiltrated metalens‐driven RIS for this application. When combined with different fluids, the resulting ultra‐compact RIS exhibits exceptional varifocal abilities, ranging from 0.4 to 0.5 mm, thereby confirming the adaptive tuning capabilities of the design. This may significantly enhance the modulation of optical waves and promote the development of RIS‐based applications in wireless communications and secure data‐transmission integrated photonic devices.

## Graphical abstract

This research investigates a fluid‐infiltrated metalens‐driven reconfigurable intelligent surface (RIS) to improve optical wireless communication (OWC) networks. RIS 1 and RIS 2 are capable of providing controlled connectivity to multiple users while concentrating on particular targets in run‐time. This study illustrates the operational mechanism of a RIS by using various isotropic liquid mediums to have wide‐range tunability in OWC.

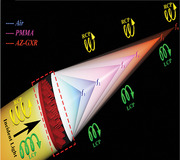

## 1 Introduction

The demand for internet bandwidth continues to grow. However, all indoor, outdoor, and satellite wireless communications currently rely on radio frequency (RF) signals.^[^ ^1^ ^]^ Due to the increasing need for higher data rates, longer distances, smaller apertures, and larger bandwidths, optical communications are considered a promising alternative and potentially superior technology compared to mature RF communications. The urgent need for advanced technology to meet the rapidly growing demands of the internet is clear. At high data rates, the directional capabilities of optical signals can be effectively utilized in indoor, underwater, and vehicle communication systems.

Reconfigurable intelligent surfaces (RIS) are regarded as a cutting‐edge technology for tuning electromagnetic (EM) signals between transmitters and receivers.^[^ ^2^, ^3^ ^]^ RIS represents a revolutionary technology in wireless communications by modifying the propagation environment to enhance signal quality and coverage. They can significantly improve spectral and energy efficiency by intelligently controlling the phase, amplitude, and polarization of incident waves, thereby enabling advanced communication paradigms for next‐generation networks. In optical wireless communication (OWC) systems, also known as free‐space optical (FSO) communications, RIS plays a crucial role in meeting key performance indicators (KPIs) like accurate positioning. Because RIS allows real‐time manipulation of input signals, making necessary adjustments for positioning quickly and without the need for moving parts. In addition, RIS effectively addresses key functionalities of FSO communication,^[^ ^4^ ^]^ such as beam steering,^[^ ^5^ ^]^ configurable wavelength division multiplexing,^[^ ^6^ ^]^ and dynamic angular diversity.^[^ ^7^ ^]^ While traditional line‐of‐sight (LoS) communication challenges are mitigated by intelligent reflecting surfaces, these structures often lack adaptive functionality, limiting their integration with advanced on‐chip devices.^[^ ^8^ ^]^ Recent innovations, however, have introduced liquid crystals into receiver designs, addressing these limitations and enabling tunable visible light communication.^[^ ^9^ ^]^

Other researchers have explored the potential of reflective surfaces for energy harvesting in visible light communication.^[^ ^10^ ^]^ Some have achieved tunability using micro‐electromechanical systems (MEMS)^[^ ^11^, ^12^ ^]^ or varactor diodes^[^ ^13^, ^14^ ^]^ to enhance RF communication capabilities. However, due to the inherently lower frequencies in the RF domain, device sizes are much larger, limiting integration with on‐chip devices. As a result, electrically‐actuated tunability^[^ ^15^ ^]^ can instead be employed by utilizing birefringent materials^[^ ^16^, ^17^ ^]^ such as liquid crystals,^[^ ^18^, ^19^, ^20^, ^21^, ^22^, ^23^ ^]^ graphene^[^ ^24^, ^25^, ^26^ ^]^ or phase‐change materials.^[^ ^27^, ^28^, ^29^ ^]^ However, there are numerous challenges associated with implementing phase‐change materials in wireless communications. These include the need to precisely adjust material properties to maintain consistent performance amid rapid temperature changes typical of both indoor and outdoor environments, as well as the potential limitations imposed by the speed of thermal processes. Moreover, liquid crystal technology also faces specific challenges, such as environmental sensitivity and response time.

Recent developments in RIS have demonstrated remarkable capabilities in wave manipulation to support diverse applications, including tunable reflective surfaces,^[^ ^30^, ^31^, ^32^ ^]^ meta‐holography,^[^ ^33^, ^34^, ^35^ ^]^ metalenses,^[^ ^36^, ^37^, ^38^, ^39^ ^]^ and beam steering.^[^ ^40^, ^41^ ^]^ The varifocal behavior of many optical technologies,^[^ ^42^ ^]^ which allows targeting specific users at precise moments, highlights the critical need for innovation in this field. While RIS technologies have primarily focused on RF signals, their potential in optical wireless communications has only recently begun to attract attention, paving the way for transformative advancements. Although considerable research has been conducted on liquid crystal‐based tunable surfaces, isotropic liquid‐based RIS has received less attention. Liquid‐transition‐based RIS offers simple fabrication, cost‐effectiveness, and a wide tunability range with smooth and continuous variations. These systems require less power, provide better thermal stability, and can be seamlessly integrated into existing systems, making them an advantageous approach for tunability mechanisms. A comparison of different tunability mechanisms and their KPIs is presented in **Table** **1**. By utilizing suitable and readily available liquids, it is possible to realize a fluid‐infiltrated metalens‐driven RIS that effectively achieves both beam steering and tunability simultaneously.

**Table 1:** Detailed analysis of KPIs of different Tunability mechanisms.

**Tunability mechanism**	**Response time**	**Cost**	**Fabrication complexity**	**Bandwidth**	**Tunability range**	**Other KPIs**	**Refs**.
MEMS	µs	Moderate	High	Wide	Moderate to high	High precision, reliability issues	[43, 44]
Graphene	ps	Low to Moderate	Moderate	Moderate	Moderate	Environmental sensitivity	[45, 46]
Phase‐change materials	ms	Moderate	Moderate	Moderate	Moderate	Nonvolatile, material degradation	[47, 48, 49]
Liquid crystal	ms to s	Low	Moderate	Narrow to Moderate	Moderate	Low power consumption	[50, 51, 52]
Fluids transition	ms	Low	Low	Wide	Wide	Continuous Tuning, Less sensitive to temperature variations	[53, 54]

**Figure 1:**
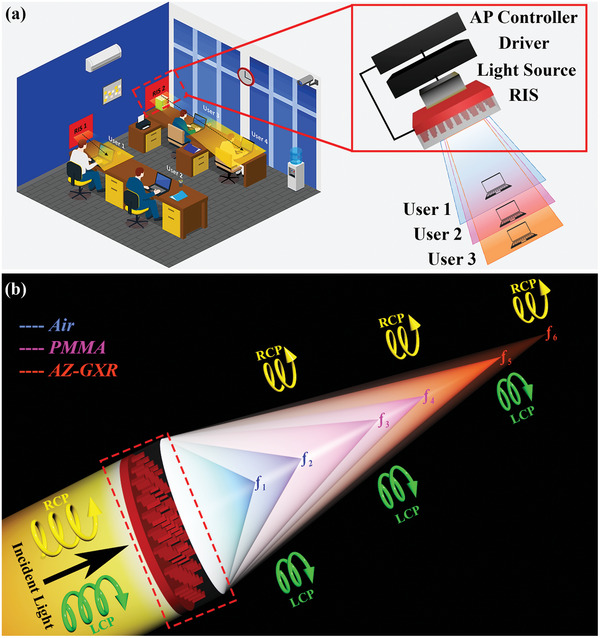
Fluid‐infiltrated metalens‐driven RIS. a) RIS‐enabled optical wireless communication environment. RIS 1 can provide controlled connectivity to user 1 and user 2 while being tuned to focus on user 1. Similarly, RIS 2 can provide connectivity to user 3 and user 4 while being tuned to focus on user 4. The inset of RIS 2 shows the envisioned functionalities of RIS‐enabled transmitter front‐ends. The designed RIS can be positioned at the front end of the transmitter and can be tuned to enhance the beam to the concerned user. b) Working principle of a fluid‐infiltrated metalens‐driven RIS. By illuminating the designed structure with input light, we get two focal points simultaneously, corresponding to left circularly polarized (LCP) and right circularly polarized (RCP) light: 𝑓_1_ and 𝑓_2_ are focal points in the case of air as a medium; 𝑓_3,_ and 𝑓_4_ are focal points in the case of PMMA; and 𝑓_5_ and 𝑓_6_ are the focal points in the case of AZ‐GXR.

This work presents a pioneering approach by introducing a fluid‐infiltrated metalens‐driven RIS designed for wide‐range tunability in OWC systems. Our proposed RIS configuration incorporates varifocal capabilities by strategically integrating various fluidic materials into an all‐silicon metasurface architecture. Our RIS enables precise adjustment of the focal point, accommodating the optical connectivity requirements of multiple aligned users. This capability, illustrated in **Figure** **1**a, is critical in complex optical communication scenarios. The figure demonstrates the adaptability of the RIS design and its ability to modify light‐matter interactions in next‐generation optical communication systems. Our study introduces an all‐silicon RIS that effectively meets key performance criteria in contemporary OWCs, including accurate positioning, robust security, and long‐term reliability. To achieve practical communication distances in meters with the proposed RIS for OWC, we can either fabricate larger metalenses or integrate conventional lenses with the existing metalens. The reconfigurability of the RIS is achieved through the use of simple isotropic liquids, offering significant versatility in manipulating the optical characteristics of the system. To demonstrate the broad tunability of the design, we engineered three distinct fluidic media as a proof of concept: air having *n*  =  1, PMMA (polymethyl methacrylate) having *n*  =   1.491, and AZ‐GXR (positive photoresist) having *n*  =  1.602, each of which enabled the generation of three distinct focal points. In addition to being efficient and compact, the RIS system is compatible with complementary metal‐oxide‐semiconductor (CMOS) technology, which significantly streamlines the fabrication process.^[^ ^41^ ^]^

## 2 Results and Discussion

A finite‐difference time‐domain (FDTD)–based numerical solver was used to design and investigate a 3D model of an RIS with a compact overall footprint of 60 µm  ×  60 µm for simulation purposes. This model consists of an array of silicon nano‐antennas on a silicon substrate, optimized for operation in the near‐infrared (NIR) range (1550 nm). The antennas are submerged in specific fluids that enable a gradual focal shift up to 3 − 5 µm, with a total focal shift of ≈10 µm occuring through all fluid transitions. In further developing this model, we also generated and experimentally examined a larger metasurface with dimensions of 0.5 mm × 0.5 mm. In the millimeter range, the experimental results of this larger‐scale prototype confirmed the desired functionality by shifting the focal plane in response to the changing infiltrated liquids.

Using the concept of a fluid‐infiltrated metalens‐driven RIS, the incident light is manipulated to constructively interfere at a targeted location. A varifocal capability of our RIS is conceptualized, with its working mechanism illustrated in Figure 1b. The image depicts an OWC environment that utilizes the fluid‐infiltrated metalens‐driven RIS, where real‐time adaptation of individual RIS units ensures accurate connections between users. The inset shows how the RIS's fluid‐infiltrated mechanism generates dual focal points for left and right circularly polarized light.

The RIS allows for the customization of multiple focal points to suit specific environments. A spin‐decoupled metalens is designed with merged phase profiles: one for left circular polarization (negative sign, *L*) and the other for right circular polarization (positive sign, *R*), as shown in Equation (1), where $r\;=\sqrt{x^{2}+y^{2}}\;$, λ defines the design wavelength, and *f* represents the focal length. There are two ways to achieve the required phase for each nano‐antenna: the Pancharatnam‐berry (PB) phase^[^ ^42^ ^]^ or the propagation phase.^[^ ^55^ ^]^ In our design, the required phase is achieved using the PB phase.

(1) $$\phi \;{\left(r,f\right)}_{L,R}=\;\mp \;\left[\frac{2\pi }{\lambda }\left(\sqrt{r^{2}+f^{2}}-f\right)\right]$$

**Figure 2:**
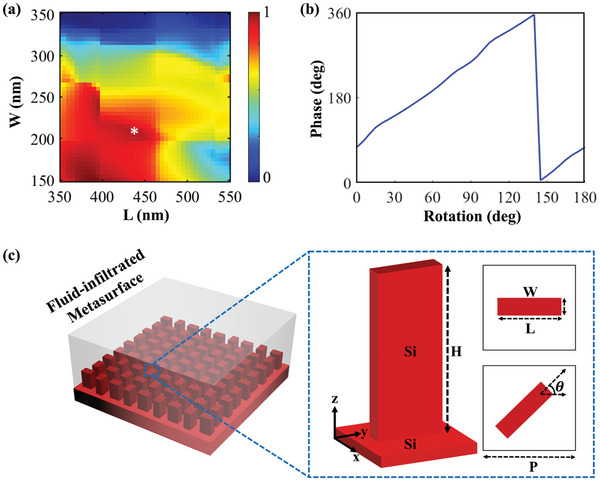
Numerical optimization and schematic diagram of the fundamental building block and designed metalens. a) Numerical simulation of the transmission of cross‐polarized light. A sweep is performed across L and W of a nano‐antenna, and the asterisk (*) sign on the plot shows selected optimized dimensions of L and W. b) Phase versus rotation. c) Schematic of a fluid‐infiltrated spin‐decoupled metalens. The nano‐antenna and the substrate are both silicon, and the height of the nano‐antenna is H  =  1240 nm. The inset also shows a top view of a unit cell, the length and width L  =  435 nm and W  =  210 nm, where the angle between the silicon nano‐antenna and the *x*‐axis is θ, and the period *p*  =  600 nm.

The fundamental building block of our fluid‐infiltrated metalens design is a bar‐shaped silicon nano‐antenna. The nano‐antenna is simulated with optimized dimensions, using periodic boundary conditions along the x and y axes, and perfectly matched layer (PML) boundary conditions along z_𝑚𝑖𝑛_ and z_𝑚𝑎𝑥_. A significant height (H) of the nano‐antennas is required to achieve complete phase coverage from 0 to 2π. The height is selected to provide an overall phase coverage of ≈4π. In our case, we chose a nano‐antenna height of 1240 nm, considering fabrication concerns such as the aspect ratio and spacing between adjacent nano‐antennas. The period (P) was then optimized for maximum transmission efficiency, ensuring that the nano‐antenna functions as a half‐wave plate with phase coverage from 0 to 2π. The optimized period is 600 nm. Additionally, the length (L) and width (W) were optimized to achieve a transmission efficiency of 70%. By rotating the nano‐antenna from 0° to 180°, a phase shift from 0° to 360° can be obtained. The selected length is 435 nm, and the width is 210 nm. The resulting nano‐antenna is shown in **Figure** **2**. The extinction coefficient (k) value is negligible at our operating wavelength, i.e., 1550 nm. The sweep results are also given in Figure 2a, showing the point of maximum transmission. With the optimized dimensions, the nano‐antenna is rotated from 0° to 180°, achieving a phase shift from 0° to 360°, as shown in Figure 2b, and the fluid‐infiltrated spin‐decoupled metalens are shown in Figure 2c. Notably, the phase modulation of the metalens is based on the PB phase by rotating nano‐antennas, which offers a significant advantage in fabrication tolerance. Even if the length and width of the nano‐antennas change after fabrication, the periodicity and rotation angle remain unchanged, so the functionalities of the metalens such as focal length and focal length change after fluid infiltration are not affected by fabrication errors (Figure S1, Supporting Information). Also, the metalens continues to function as a half‐wave plate because the transmission of cross‐polarized light remains high, despite variations in the length and width of the silicon nano‐antennas.

**Figure 3:**
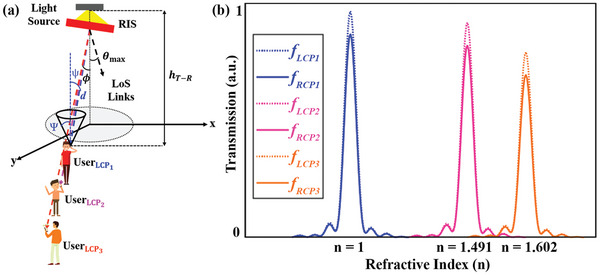
Optical wireless communication channel model via Lambertian radiation pattern. a) A transmission scenario model of RIS providing multiple users with point‐to‐point OWC downlink connectivity. Combining the designed metasurface with various liquids makes it possible to manipulate the refractive index (*n*  =  1.0 − 1.602) of the nano‐antenna surroundings, and consequently, the focal length in real‐time. RIS denotes the fluid‐infiltrated metalens‐driven RIS, RCP denotes right circularly polarized light, LCP denotes left circularly polarized light, and LoS denotes line‐of‐sight. b) Performance simulation of a nano‐antenna optimized for transmitting cross‐polarized diffracted light when incorporated into three distinct fluidic materials.

The primary application of OWC is to establish LoS connections between indoor transmitters and receivers.^[^ ^56^ ^]^ Although a substantial body of literature discusses Non‐Line‐of‐Sight (NLoS) scenarios in OWC channels,^[^ ^57^ ^]^ the LoS signal is significantly superior to any reflected signal. Because the reflected signals in NLoS scenarios suffer from higher losses and distortions, reducing both signal strength and reliability. A LoS link, however, requires a reliable and precise OWC positioning system. Therefore, we will focus on the characteristics of LoS channels in an OWC example. The DC gain of the optical channel, as shown in **Figure** **3**, which connects a light source transmitter and a photodetector (PD), can be calculated using the Lambertian radiation pattern as described below^58^ ^]^:

(2) $$\mathrm{Gai}n_{T-R}\left(0\right)=\left\{\begin{array}{c} \frac{\left(m_{L}+1\right)A_{\mathrm{PD}}}{2\pi d^{2}}\cos^{m_{L}}\left(\phi \right)\cos\left(\psi \right),\psi \leq x\leq \Psi \\ 0,\psi >\Psi \end{array}\right.$$

In Equation (2), the Lambert index of the light source is given as *m_L_* = −1/log _2_[cos *θ*_max_] , where the *θ*_max_ represents the source radiation semi‐angle at half power of the light. Additionally, ψ and *ϕ* define the angles of incidence and irradiance, respectively. Ψ refers to the field‐of‐view semi‐angle of the photodetector (PD), which has an effective area of A_PD_. The maximum range of this OWC system is defined as:

(3) $$\;\;r=h_{T-R}\times \tan\left(\Psi \right)$$

In Equation (3), *h*_*T* − *R*_ is the height, and in Equation (2), *d* is the distance between the transmitter and receiver. If the receiver is outside this maximum range, it may not receive any signal from the light source, resulting in an output DC value of 0 on the receiving side. A transmission scenario model of a RIS enabling point‐to‐point OWC downlink connectivity for multiple users is shown in Figure 3a. For tuning purposes, a slab is placed on top of the structured nano‐antennas, and to shift the focal point, the material in the slab is varied from air to other fluids having an n greater than unity. As a proof of concept, we selected three focal point variations: focal point 𝑓_1_ in the presence of air, focal point 𝑓_2_ in the presence of a layer of PMMA, and focal point 𝑓_3_ in the presence of a layer of AZ‐GXR. The slab thickness is set to 20 µm. Figure 3b shows the simulated transmitted intensities for all three liquids.

All numerical simulations were performed using FDTD Solutions from Lumerical Inc. Initially, during optimization, we considered a unit cell containing a bar‐shaped silicon nano‐antenna. The unit cell was simulated with periodic boundary conditions in the x and y directions, with a period of 600 nm in both directions. The light was shone from the bottom along the *z*‐direction, and a slab with varying refractive indices represented the fluid. The placement of the nano‐antennas was based on merged phase profiles, with designed focal lengths of 𝑓_𝐿𝐶𝑃_ = 30 µm and 𝑓_𝑅𝐶𝑃_ = 40 𝜇m. After positioning all the nano‐antennas, a complete surface area of 60 µm × 60 µm was simulated. The minimum mesh size in the simulations was set to less than λ/10, i.e., 100 nm. The designed fluid‐infiltrated metalens‐driven RIS operates with either LCP or RCP incident light, as all of the nano‐antennas contribute to the phase delay in both cases. Initially, the metasurface was simulated with LCP incident light without any fluid, and the resulting focal length 𝑓_1_ was 30.50 µm, as shown in Figure S2b (Supporting Information). Next, the metasurface was immersed in a 20 µm‐thick layer of PMMA, and the simulated focal length 𝑓_2_ was 36.50 µm as shown in Figure S2d (Supporting Information). Finally, the metasurface was immersed in a 20 µm‐ thick layer of AZ‐GXR, resulting in a focal length 𝑓_3_ of 39.70 µm as shown in Figure S2f (Supporting Information). All xy‐plane surface plots of the cross‐polarized light are displayed in Figure S2a,c,e (Supporting Information), while the xz‐plane surface plots are shown in Figure S2b,d,f (Supporting Information).

**Figure 4:**
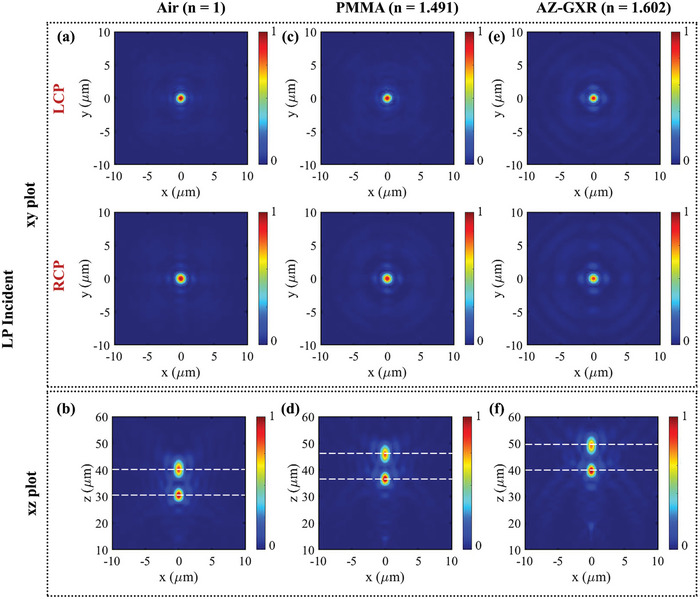
Simulated results of the fluid‐infiltrated metalens‐driven RIS with linearly polarized incident light. a,b) Surface plots (xy: |𝐸_𝐿𝐶𝑃_|^2^ and |𝐸_𝑅𝐶𝑃_|^2^ and xz: |𝐸|^2^) of RIS infiltrated with air. c,d) Surface plots (xy: |𝐸_𝐿𝐶𝑃_|^2^ and |𝐸_𝑅𝐶𝑃_|^2^ and xz: |𝐸|^2^) of RIS infiltrated with PMMA. e,f) Surface plots (xy: |𝐸_𝐿𝐶𝑃_|^2^ and |𝐸_𝑅𝐶𝑃_|^2^ and xz: |𝐸|^2^) of RIS infiltrated with AZ‐GXR.

It is observed that as the refractive index of the fluid increases, both the focal length and the focal plane shift further. This RIS can be deployed in OWC applications where multiple users are within the LoS of the RIS, allowing light to be directed to any user by tuning the RIS. First, the RIS was simulated with RCP incident light without any fluid, and the achieved focal length 𝑓_1_ was 40.20 µm, as shown in Figure S3b (Supporting Information). Next, the RIS was immersed in a 20 µm‐thick layer of PMMA, resulting in a simulated focal length 𝑓_2_ of 45.80 µm as shown in Figure S3d (Supporting Information). Finally, the RIS was immersed in a 20 µm‐thick layer of AZ‐GXR, shifting the simulated focal length 𝑓_3_ to 48.90 µm as shown in Figure S3f (Supporting Information). The xy‐plane surface plots of the cross‐polarized light are shown in Figure S3a,c,e (Supporting Information), while the xz‐plane surface plots are displayed in Figure S2b,d,f (Supporting Information). The RIS was then simulated with linearly polarized incident light, without any fluid at first, resulting in two focal lengths corresponding to LCP and RCP: 𝑓_𝐿𝐶𝑃_ at 30.40 µm and 𝑓_𝑅𝐶𝑃_ at 40.20 µm, as shown in **Figure** **4**b. After immersing the RIS in a 20 µm‐thick layer of PMMA, the simulated focal lengths shifted to 𝑓_𝐿𝐶𝑃_ at 36.60 µm and 𝑓_𝑅𝐶𝑃_ at 45.80 µm as shown in Figure 4d. Finally, when immersed in a 20 𝜇m‐thick layer of AZ‐GXR, the focal lengths shifted further to 𝑓_𝐿𝐶𝑃_ at 39.70 µm and 𝑓_𝑅𝐶𝑃_ at 49.00 µm, as shown in Figure 4f. The xy‐plane surface plots of |𝐸_𝐿𝐶𝑃_|^2^ and |𝐸_𝑅𝐶𝑃_|^2^ are presented in Figure 4a,c,e, while the xz‐plane surface plots of |𝐸|^2^ are presented in Figure 4b,d,f.

**Figure 5:**
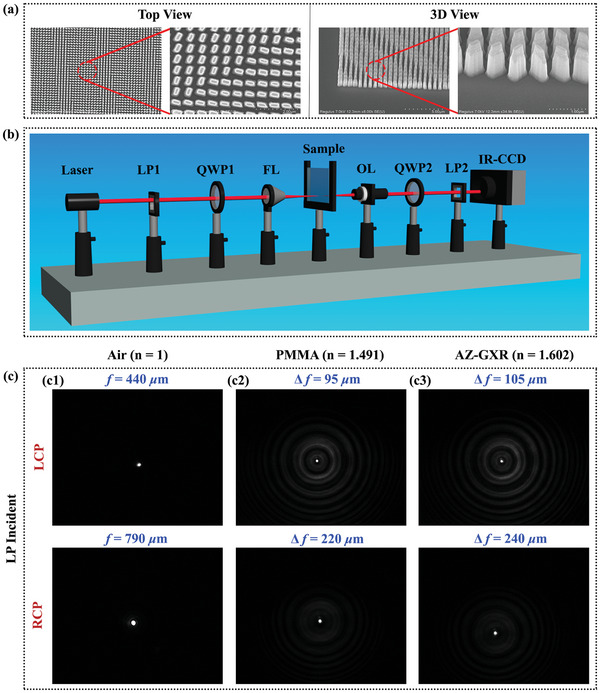
Fabrication and characterization of the RIS. a) SEM images of the fabricated metalens for efficient optical wireless communications (The left side shows the top view and the right side shows the 3D view of the fabricated metalens). b) Schematic of the optical setup of the fabricated metalens infiltrated with the fluid. NIR laser was used as the light source. Here, LP = linear polarizer, QWP = quarter‐wave plate, FL = focusing lens, OL = objective lens, IR‐CCD = infrared charge‐coupled device c) Experimental results of the focal spots for three different media.

**Figure** **5**a shows various views of the scanning electron microscopy (SEM) images of the designed structure, with the fabricated metalens having an overall size of 0.5 mm  ×  0.5 mm. Figure 5b presents a custom‐built setup for optical microscopy. A laser source with a wavelength of 1550 nm was used to generate the input beam, which then passes through a linear polarizer and a quarter‐wave plate to achieve the desired handedness of circularly polarized light. Next, a focusing lens was used to focus the circularly polarized light on the fluid‐infiltrated metalens. The diffracted light from the metalens impinges on the objective lens, which magnifies the visible intensity distribution. The focusing lens and objective lens are aligned such that the device under test is positioned at the intersection of their focal planes, allowing collimated light to be obtained from the objective lens. A combination of a quarter‐wave plate and linear polarizer was then used to filter specific polarization at the output. Finally, the transmitted beam is recorded using an infrared charge‐coupled device (IR‐CCD). To determine the focal lengths of the metalens, we used a standard micrometer and a translation stage equipped with the objective lens, quarter‐wave plate 2, linear polarizer 2, and the IR‐CCD. First, we captured the image of the metalens surface, then repositioned the objective lens to capture the image of the focal point. The focal length was calculated by subtracting the micrometer readings between these two positions.

Initially, the results were observed for the air medium, with the focal length for LCP at 440 µm, and the focal length for RCP at 790 µm, as shown in Figure 5c, 1. Then, a 1 µm layer of PMMA 495 A8 was spin‐coated onto the metalens at 1000 rpm twice. A focal length shift of 95 µm was observed for the LCP focal length, and a focal length shift of 220 µm was observed for the RCP focal length as shown in Figure 5c, 2. Afterward, the PMMA layer was removed by dipping the metalens in acetone for 15 min at 75 °C, followed by sonication at 45 Hz for 4 min same as the lift‐off process. Then a 2 µm layer of AZ GXR‐601 46cp was spin‐coated onto the metalens at 5000 rpm. As a result, a focal length shift of 105 µm was observed for the LCP focal length, and a focal length shift of 240 µm was observed for the RCP focal length, as shown in Figure 5c, 3. It was observed that as the refractive index of the infiltrated medium increased from *n*  =  1 to a higher value, the focal length shifted upward, and the overall focal variation also increased. This variation in focal length can potentially be utilized for a tunable architecture in FSO communication.

## 3 Conclusion

This study introduces a design methodology of a fluid‐infiltrated metalens‐driven RIS to achieve wide‐range tunability of focal variation. The developed fluid‐infiltrated metalens‐driven RIS enables secure data transmission to a specific user in a line of multiple users. To demonstrate the varifocal behavior, three different fluidic media were used as a proof of concept: air with a refractive index of 1, PMMA with a refractive index of 1.491, and AZ‐GXR with a refractive index of 1.602. The simulated focal lengths with three different fluidic media exhibited a range of 30.5 − 39.70 µm when illuminated with LCP light and from 40.20 to 48.90 µm when illuminated with RCP light. Given the design's compatibility with CMOS technology and ease of fabrication, a larger metasurface measuring 0.5 mm  ×  0.5 mm was produced and experimentally validated. The exceptional varifocal capabilities of the fabricated ultra‐compact RIS, ranging from 0.4 to 0.5 mm, confirm the design's adaptive tuning abilities. The wide‐range focal variation capability of the proposed design not only sets a new precedent but also encourages the development of more complex design methods such as deep learning^[^ ^59^, ^60^, ^61^ ^]^ and scalable optical devices based on our RIS methodology.

## 4 Experimental Section

### Fabrication of Metalens

The silicon metalens were fabricated using standard EBL (ELIONIX, ELS‐7800; acceleration voltage: 80 kV, beam current: 100 pA). First, the positive tone electron beam resist of polymethyl methacrylate (MicroChem, 495 PMMA A6) was spin‐coated on a double‐side polished silicon substrate at 2000 rpm for 60 s. The spin‐coated resist was baked at 180 °C for 5 min. Next, the substrate was cooled for 90 s, followed by spin‐coating of a conductive polymer layer (Showa Denko, E‐spacer 300Z) on top of the electron beam resist layer at 2000 rpm for 60 s to prevent the charging effect from the dielectric layer. After the exposure process, the conductive layer was removed using de‐ionized (DI) water, and the electron beam resist was developed in methyl isobutyl ketone (MIBK)/isopropyl alcohol (IPA) 1:3 solutions for 15 min. A 120 nm thick Cr mask layer was deposited using an electron‐beam evaporator (KVT, KVE‐ENS4004), followed by a lift‐off process of dipping in acetone for 15 min at 75 °C and a sonication process of 45 Hz for 4 min. Finally, Cr mask patterns were transferred onto the double‐side polished silicon substrate using a dry etching process (DMS, silicon/metal hybrid etcher), followed by the remover of Cr mask using Cr etchant (CR‐7).

## Conflict of Interest

The authors declare no conflict of interest.

## Supporting information



Supporting Information

## Data Availability

The data that support the findings of this study are available from the corresponding author upon reasonable request.
