# Supplementary material for: Fluid‐Infiltrated Metalens‐Driven Reconfigurable Intelligent Surfaces for Optical Wireless Communications
Source: Adv Sci (Weinh). 2024 Sep 28;11(43):2406690. doi: 10.1002/advs.202406690 (PMC11578380; doi:10.1002/advs.202406690)
Supplement: Supplementary file 1 — Supporting Information [file ADVS-11-2406690-s001.docx]

Supporting Information

**Fluid-Infiltrated Metalens-Driven Reconfigurable Intelligent Surfaces for Optical Wireless Communications**

Ramna Khalid^1†^_,_ Jaekyung Kim^2†^, Nasir Mahmood^1†^, Humberto Cabrera^3^, Muhammad Qasim Mehmood^1^*, Aaron Danner^4^*, Muhammad Zubair^1^*, Junsuk Rho^2,5,6,7,8^*

Ramna Khalid, Nasir Mahmood, Prof. Muhammad Qasim Mehmood, Prof. Muhammad Zubair

^1^MicroNano Lab, Department of Electrical Engineering, Information Technology University of the Punjab (ITU), 54000 Lahore, Pakistan

* E-mail: qasim.mehmood@itu.edu.pk, muhammad.zubair@itu.edu.pk

Jaekyung Kim, Prof. Junsuk Rho

^2^Department of Mechanical Engineering, Pohang University of Science and Technology (POSTECH), Pohang 37673, Republic of Korea

* E-mail: jsrho@postech.ac.kr

Dr. Humberto Cabrera

^3^MLab, STI Unit, The Abdus Salam International Centre for Theoretical Physics, 34151 Trieste, Italy

Prof. Aaron Danner

^4^Department of Electrical and Computer Engineering National University of Singapore 4 Engineering Drive 3, Singapore 117583, Singapore

* E-mail: adanner@nus.edu.sg

Prof. Junsuk Rho

^5^Department of Chemical Engineering, Pohang University of Science and Technology (POSTECH), Pohang 37673, Republic of Korea

Prof. Junsuk Rho

^6^Department of Electrical Engineering, Pohang University of Science and Technology (POSTECH), Pohang 37673, Republic of Korea

Prof. Junsuk Rho

^7^POSCO-POSTECH-RIST Convergence Research Center for Flat Optics and Metaphotonics, Pohang 37673, Republic of Korea

Prof. Junsuk Rho

^8^National Institute of Nanomaterials Technology (NINT), Pohang 37673, Republic of Korea

**^†^ These authors have contributed equally to this work.**

Keywords: varifocal metalens, reconfigurable metalens, intelligent metasurface, fluid-infiltration, optical communication


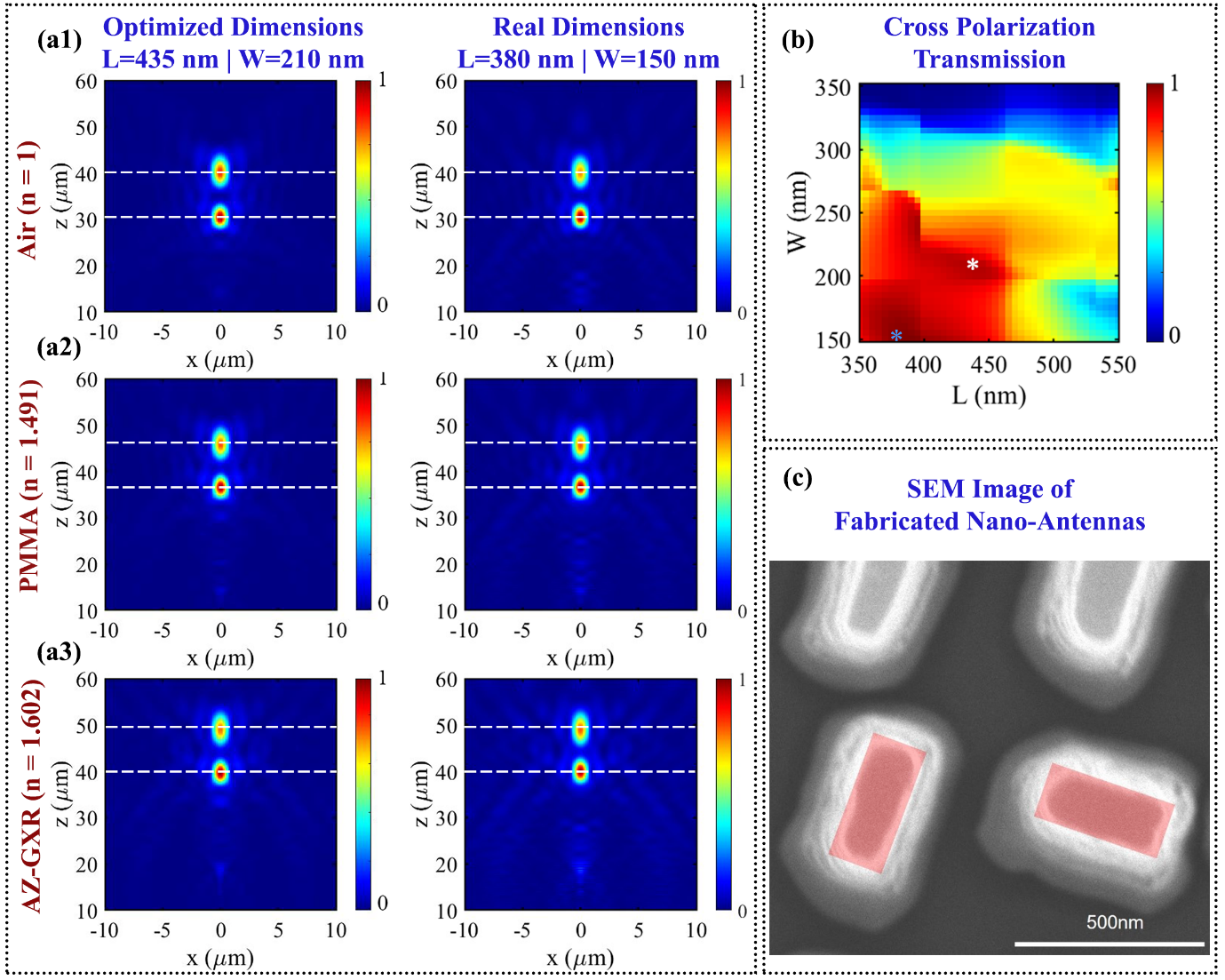


**Figure S1.** Fabrication tolerance of the fluid-infiltrated spin-decoupled metalens. (a1-a3) Simulated results of the focal length change of the metalens between optimized dimensions and the real dimensions for three different media: Air, PMMA, and AZ-GXR, respectively. (b) Transmission of the cross-polarized light depicts high transmission for both the optimized and real dimensions. (c) SEM image of the nano-antennas ($length=380 nm$, $width=150 nm$) after fabrication.


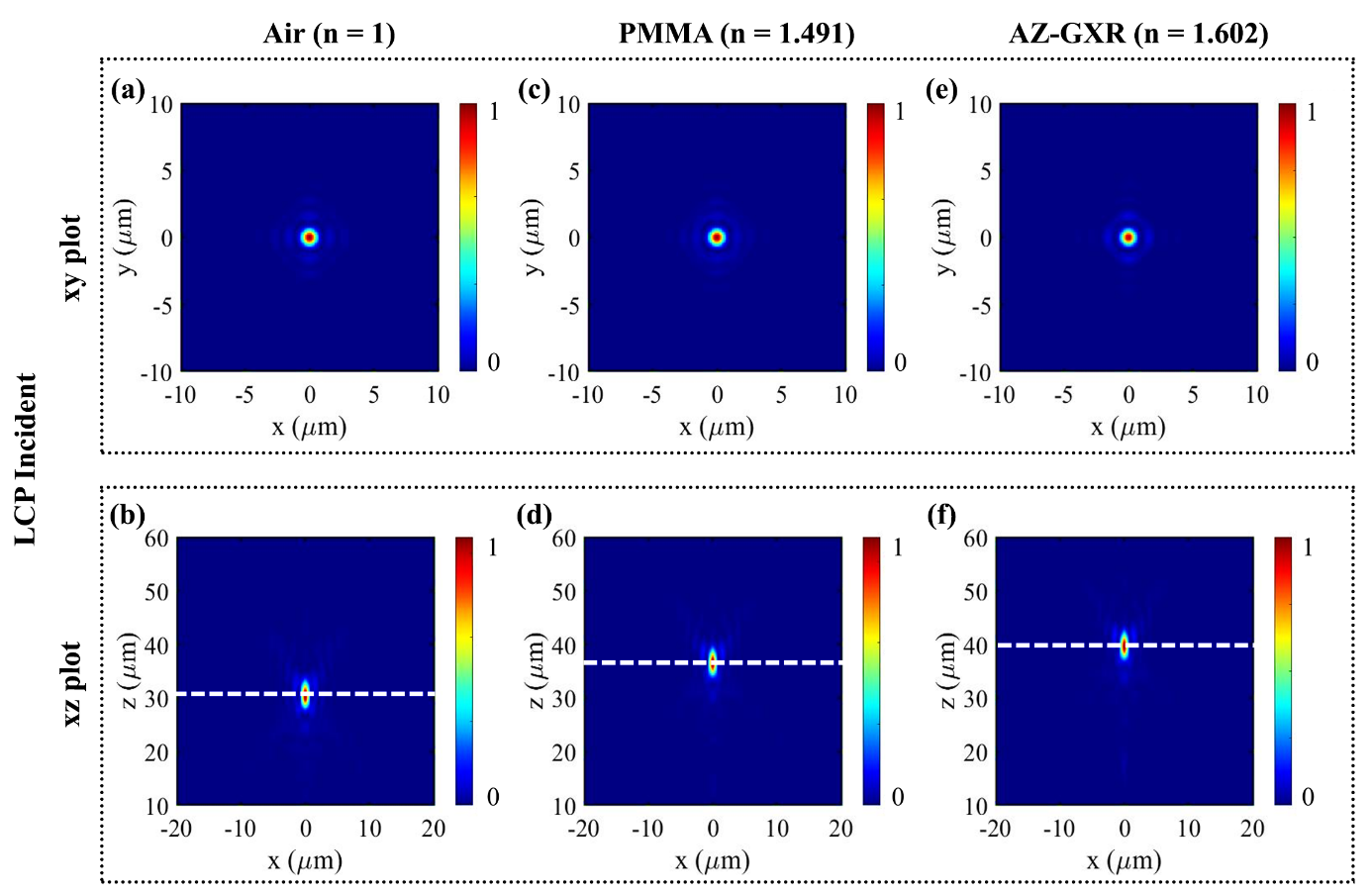


**Figure S2.** Simulated results of RIS with left circularly polarized incident light. (a, b) Surface plots (cross-polarized light: xy and xz) of RIS infiltrated with air. (c, d) Surface plots (cross-polarized Light: xy and xz) of RIS infiltrated with PMMA. (e, f) Surface plots (cross-polarized Light: xy and xz) of RIS infiltrated with AZ-GXR.


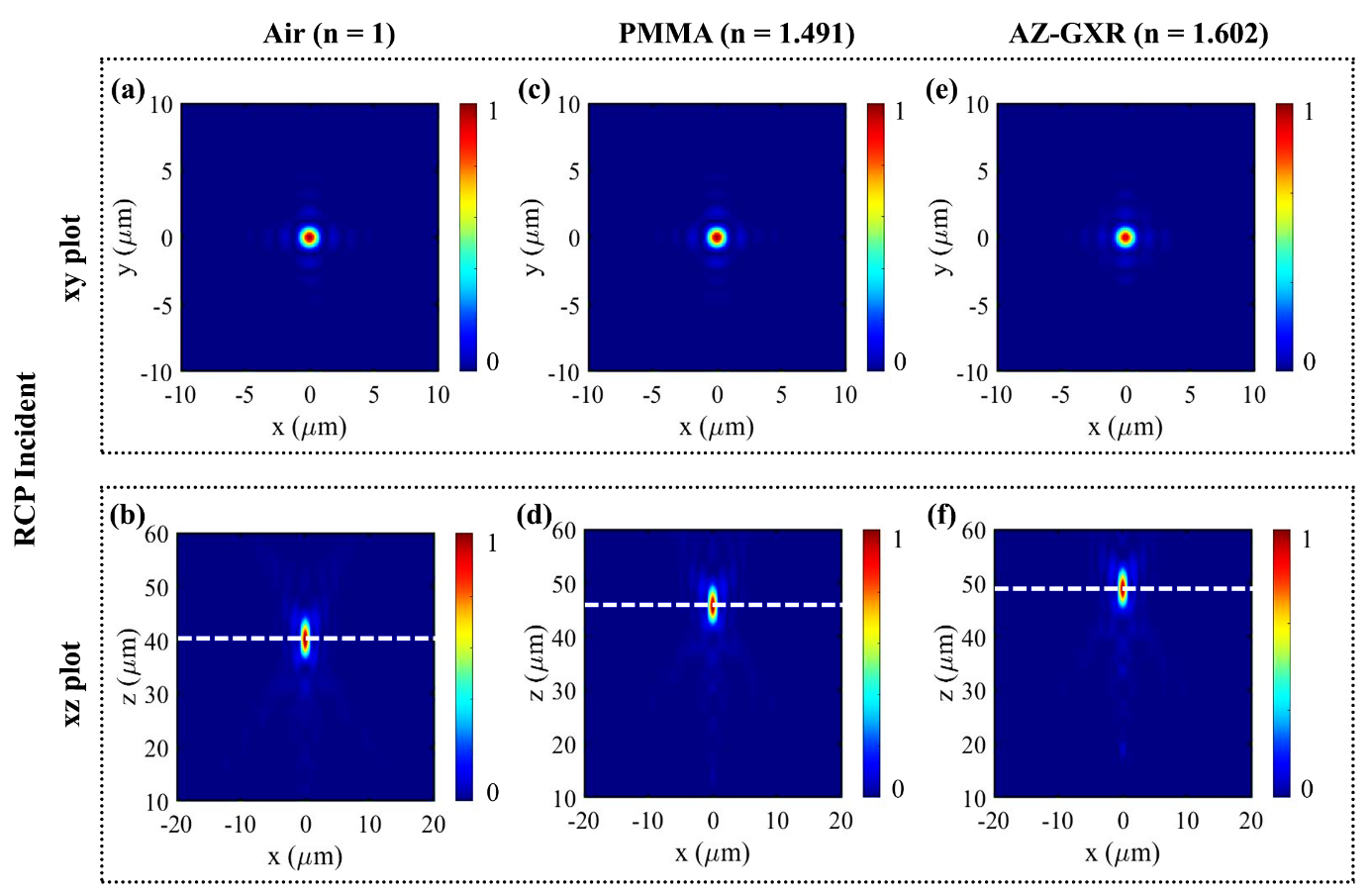


**Figure S3**. Simulated results of RIS with right circularly polarized incident light. (a, b) Surface plots (cross-polarized light: xy and xz) of RIS infiltrated with air. (c, d) Surface plots (cross-polarized Light: xy and xz) of RIS infiltrated with PMMA. (e, f) Surface plots (cross-polarized Light: xy and xz) of RIS infiltrated with AZ-GXR.
